# Supplementary material for: Autistic traits, but not schizotypy, predict increased weighting of sensory information in Bayesian visual integration
Source: eLife. 2018 May 14;7:e34115. doi: 10.7554/eLife.34115 (PMC5966274; doi:10.7554/eLife.34115)
Supplement: Transparent reporting form [file elife-34115-transrepform.pdf]

eLIFE
1st Floor
24 Hills Road
Cambridge CB2 1JP, UK
P 01223 855340
W elifesciences.org
T @elife

eLife's transparent reporting form

We encourage authors to provide detailed information *within their submission* to facilitate the interpretation and replication of experiments. Authors can upload supporting documentation to indicate the use of appropriate reporting guidelines for health-related research (see EQUATOR Network), life science research (see the BioSharing Information Resource), or the ARRIVE guidelines for reporting work involving animal research. Where applicable, authors should refer to any relevant reporting standards documents in this form.

If you have any questions, please consult our Journal Policies and/or contact us: editorial@elifesciences.org.

Sample-size estimation

- You should state whether an appropriate sample size was computed when the study was being designed
- You should state the statistical method of sample size computation and any required assumptions
- If no explicit power analysis was used, you should describe how you decided what sample (replicate) size (number) to use

Please outline where this information can be found within the submission (e.g., sections or figure legends), or explain why this information doesn't apply to your submission:

Since this is the first time this task is used to look at correlations with personality traits, we were not sure what effect size to look for, and on which of our several experimental measures such effect could manifest. We tried to get as many participants as possible during the course of the project (roughly one year of data collection) and so that we had a sample size larger than related studies in this area. We have 83 participants, while related studies looking at correlations with AQ had 29-57 participants (Skewes et al 2014: 29 participants, Powell et al 2016: 31 participants; Sevgi et al 2016: 36 participants; Lawson et al 2017: 57 participants).

Replicates

- You should report how often each experiment was performed
- You should include a definition of biological versus technical replication
- The data obtained should be provided and sufficient information should be provided to indicate the number of independent biological and/or technical replicates
- If you encountered any outliers, you should describe how these were handled
- Criteria for exclusion/inclusion of data should be clearly stated
- High-throughput sequence data should be uploaded before submission, with a private link for reviewers provided (these are available from both GEO and ArrayExpress)

Please outline where this information can be found within the submission (e.g., sections or figure legends), or explain why this information doesn't apply to your submission:

eLife Sciences Publications, Ltd is a limited liability non-profit non-stock corporation incorporated in the State of Delaware, USA, with company number 5030732, and is registered in the UK with company number FC030576 and branch number BR015634 at the address 1st Floor, 24 Hills Road, Cambridge CB2 1JP | August 2014

The criteria for exclusion are based on performance criteria that were determined prior to / also used in our previously published study (Chalk et al 2010). They are described in the Methods section and in supplementary Figure 1. The main statistical learning effects that we are looking at were previously described in Chalk et al (2010) and Gekas et al (2015).

Statistical reporting

- Statistical analysis methods should be described and justified
- Raw data should be presented in figures whenever informative to do so (typically when N per group is less than 10)
- For each experiment, you should identify the statistical tests used, exact values of N, definitions of center, methods of multiple test correction, and dispersion and precision measures (e.g., mean, median, SD, SEM, confidence intervals; and, for the major substantive results, a measure of effect size (e.g., Pearson's r, Cohen's d)
- Report exact p-values wherever possible alongside the summary statistics and 95% confidence intervals. These should be reported for all key questions and not only when the p-value is less than 0.05.

Please outline where this information can be found within the submission (e.g., sections or figure legends), or explain why this information doesn't apply to your submission:

We have presented the raw data in the figures, and described the statistical tests used. P values are reported as well as 95% confidence intervals throughout the manuscript.

(For large datasets, or papers with a very large number of statistical tests, you may upload a single table file with tests, Ns, etc., with reference to sections in the manuscript.)

Group allocation

- Indicate how samples were allocated into experimental groups (in the case of clinical studies, please specify allocation to treatment method); if randomization was used, please also state if restricted randomization was applied
- Indicate if masking was used during group allocation, data collection and/or data analysis

Please outline where this information can be found within the submission (e.g., sections or figure legends), or explain why this information doesn't apply to your submission:

This doesn't apply to our submission since we focus on correlation measures between task performance and personality traits (questionnaire scores), rather than (potentially artificially constructed) group differences.

Additional data files ("source data")

- We encourage you to upload relevant additional data files, such as numerical data that are represented as a graph in a figure, or as a summary table
- Where provided, these should be in the most useful format, and they can be uploaded as "Source data" files linked to a main figure or table
- Include model definition files including the full list of parameters used
- Include code used for data analysis (e.g., R, MatLab)
- Avoid stating that data files are "available upon request"

Please indicate the figures or tables for which source data files have been provided:

We upload additional data files regarding our participants' questionnaire scores, estimation, discrimination performance as csv files and the Matlab code that leads to replication of Figures 3a-f, Figure 2b, Figure 7. All data is fully anonymised.
